# Dietary amino acid and vitamin complex protects honey bee from immunosuppression caused by *Nosema ceranae*

**DOI:** 10.1371/journal.pone.0187726

**Published:** 2017-11-08

**Authors:** Uros Glavinic, Biljana Stankovic, Vladimir Draskovic, Jevrosima Stevanovic, Tamas Petrovic, Nada Lakic, Zoran Stanimirovic

**Affiliations:** 1 Department of Biology, Faculty of Veterinary Medicine, University of Belgrade, Belgrade, Serbia; 2 Laboratory for Molecular Biomedicine, Institute of Molecular Genetics and Genetic Engineering, University of Belgrade, Belgrade, Serbia; 3 Scientific Veterinary Institute “Novi Sad”, Novi Sad, Serbia; 4 Department of Statistics, Faculty of Agriculture, University of Belgrade, Belgrade, Serbia; International Nutrition Inc, UNITED STATES

## Abstract

Microsporidium *Nosema ceranae* is well known for exerting a negative impact on honey bee health, including down-regulation of immunoregulatory genes. Protein nutrition has been proven to have beneficial effects on bee immunity and other aspects of bee health. Bearing this in mind, the aim of our study was to evaluate the potential of a dietary amino acid and vitamin complex “BEEWELL AminoPlus” to protect honey bees from immunosuppression induced by *N*. *ceranae*. In a laboratory experiment bees were infected with *N*. *ceranae* and treated with supplement on first, third, sixth and ninth day after emergence. The expression of genes for immune-related peptides (abaecin, apidaecin, hymenoptaecin, defensin and vitellogenin) was compared between groups. The results revealed significantly lower (p<0.01 or p<0.001) numbers of *Nosema* spores in supplemented groups than in the control especially on day 12 post infection. With the exception of abacein, the expression levels of immune-related peptides were significantly suppressed (p<0.01 or p<0.001) in control group on the 12^th^ day post infection, compared to bees that received the supplement. It was supposed that *N*. *ceranae* had a negative impact on bee immunity and that the tested amino acid and vitamin complex modified the expression of immune-related genes in honey bees compromised by infection, suggesting immune-stimulation that reflects in the increase in resistance to diseases and reduced bee mortality. The supplement exerted best efficacy when applied simultaneously with *Nosema* infection, which can help us to assume the most suitable period for its application in the hive.

## Introduction

*Nosema* spp. microsporidia frequently parasitize adult honey bees [1]. Two species have been described: *Nosema apis* and *N*. *ceranae*. Numerous reports revealed that *N*. *ceranae* predominates over *N*. *apis*, as much greater occurrence and much wider geographical distribution of the former have been evidenced in most parts of the world [1–9]. *N*. *ceranae* infection impacts both individual honey bees [4,10–12] and the colony, and has been associated with colony collapse disorder—CCD [10,13–16] influencing the reproduction and productivity of honey bee colony [17,18].

Insects have a robust immune system to defend themselves against the attack of different pathogens. This system includes physical barriers as the first line of defense, and innate cellular and humoral immunity -a second line of defence [19]. Antimicrobial peptides apidaecin [20], abaecin [21], hymenoptaecin [22] and defensin [23], components of humoral immunity, contribute a lot to the defense against microorganisms [24]. Honey bee vitellogenin is a female-specific yolk protein, which is recognized as one of the most important regulators of immunity and longevity of honey bees [25, 26]. Expressions of genes that code for these immune-related proteins were investigated in several studies: Antúnez et al. [24] demonstrated that expressions of some genes in *N*. *ceranae* infected bees were significantly down-regulated, and Chaimanee et al. [27] reaffirmed host immunosuppression by *N*. *ceranae* infection. Other pathogens, for example *Varroa destructor* [28], viruses [29,30] and *Paenibacillus larvae* [31], also affect honey bee immunity. Furthermore, some results revealed the positive impact of nutrition on certain aspects of bee immunity [32,33], including those of pollen nutrients on genes affecting longevity and the production of some antimicrobial peptides [34,35].

Diet supplementation in beekeeping practice is common in cases of natural forage deficiency. Unfortunately, recent decades have witnessed considerable loss of natural habitats that have inevitably led to the reduction in floral abundance and diversity [36]. As a consequence, a shortage of natural bee forage (pollen and nectar) appears in many regions and triggers the need of adequate supplemental diets [37,38] that may reduce colony losses by alleviating protein stress [39]. The most common supplements for honey bees are those based on amino acids and vitamins, and one of them is "BEEWELL AminoPlus". It has been widely used in the Balkan countries from 2010, but scientific investigations into its influence on bees have been yet to be done.

Numerous studies evidenced the beneficial role of proteins from pollen in physiological processes, brood rearing, adult population growth and production of royal jelly [39–42]. Natural proteins in honey bee nutrition are essential for maintaining colony fitness because they positively affect colony health, immune response, parasite tolerance and survival [32,39,43–45], worker longevity [35] and the reproductive quality of drones [46]. Natural bee-made protein-rich diet (bee bread) originating from pollen, especially polyfloral, is the best natural source of proteins and vitamins for honey bees [37,44,45]. When it comes to protein supplements (artificial high-protein diets containing no pollen), their effects on bees are variable and dependent on composition and/or formulation. Three different pollen-free commercial diets manifested better, comparable or worse effects than pollen cake in terms of stimulating brood rearing and/or adult population growth [42]. No significant influence on brood and colony development, winter survival and productive capacity (pollen and honey reserves) was caused by protein/vitamin supplementation in the study of Pajuelo et al. [47]. Among three protein supplements, FeedBee^®^ (non-soy-based), BeePro^®^ (soy-based) and TLS Bee Food, only Feed-Bee^®^ displayed the same effects as pollen on brood rearing, colony growth and honey production, while the efficacy of the other two was weaker [48].

There is also no consistency among results of studies where protein titer in the haemolymph of caged honey bees was used as marker of protein diet efficacy: De Jong et al. [49] found protein supplements FeedBee^®^ and BeePro^®^ more efficient than natural pollen; Morais et al. [50] observed no differences among artificial self-made protein-rich diets and bee bread; DeGrandi-Hoffman et al. [43] reported no differences between the effects of BeePro^®^, MegaBee^®^ and *Brassica rapa* pollen; and Basualdo et al. [45] reported significantly higher haemolymph protein titres, but also better survival in bees fed with bee bread than those fed with the substitute.

Inconsistent results were also reported considering the effects of protein diets on pathogen levels. In work of Basualdo et al. [45] higher *N*. *ceranae* abundance (but also survival) was recorded in bees fed with bee bread compared to those whose diet was supplemented with a substitute (artificial protein diet Virgen^®^); conversely, DeGrandi-Hoffman et al. [43] reported higher pathogen (*Nosema* and virus) levels and more serious queen and colony losses in groups fed with supplements, especially with BeePro^®^.

Starting from previous findings of *N*. *ceranae* suppressive effects on immune-related genes [24,27] and beneficial effects of adequate protein nutrition on honey bee colony health and development, immune response, parasite tolerance and survival [32–34,39,43–45,51] we hypothesized that “BEEWELL AminoPlus” (a dietary supplement very rich in amino acids and vitamins) could influence the honey bee immunity. Thus, the aim of this study was to assess the potential of this supplement to protect honey bees compromised by *N*. *ceranae* infection.

## Materials and methods

### Tested supplement

A mixture of vitamins, minerals and amino acids (Table 1), which is sold under the brand name “BEEWELL AminoPlus” (Provet, Ankara, Turkey) was tested in this study. The feeding solution was prepared according to the manufacturer's instructions (1 ml of BEEWELL AminoPlus/1 l of sugar syrup).

**Table 1:** Composition of 1 liter of “BEEWELL AminoPlus”.

Compound	Qty	Compound	Qty	Compound	Qty	Compound	Qty
Tryptophan	2.0 g	Proline	15.0 g	Valine	5.5 g	Vitamin B12	0.001 g
Hydroxyproline	15.0 g	Alanine	15.0 g	Isoleucine	5.4 g	Vitamin B6	0.2 g
Glutamic Acid	15.0 g	Arginine	15.0 g	Threonine	3.0 g	Vitamin B2	0.8 g
Asparagine	7.0 g	Histidine	1.0 g	DLMethionine	2.5 g	Vitamin B1	0.2 g
Phenylalanine	1.5 g	Glycine	30.0 g	L-Lysine	10.0 g	Vitamin E	0.28 g
Hydroxylysine	1.2 g	Serine	5.0 g	Vitamin K3	1.5 g	Vitamin D3	270.000 IU
Tyrosine	0.6 g	Leucine	6.0 g	Vitamin C	3.0 g	Vitamin A	1.800.000 IU
Ca-D-Pantothenate	1.2 g	Niacin	2.4 g				

### Experimental design

**Fig 1:**
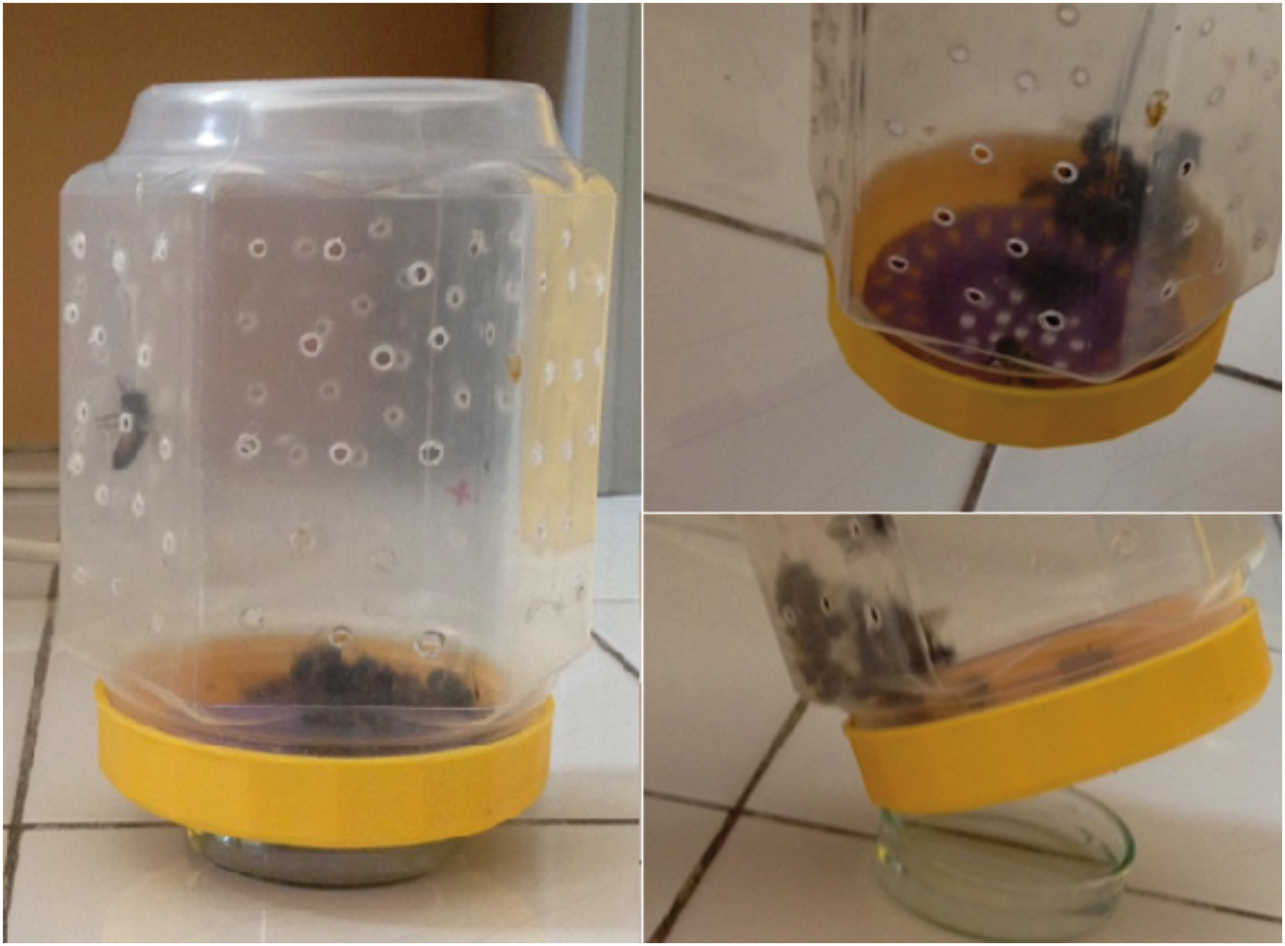
The modification of a laboratory cage: A plastic jar with small holes made for aeration and a plastic mesh sink strainer inserted into the lid allowing bees to take the food from a small petri dish placed below.

Brood for the experiment was taken from a healthy *Apis mellifera* colony belonging to the experimental apiary of the Faculty of Veterinary Medicine, University of Belgrade. The colony was *Nosema*-free, as confirmed by PCR, using methodology described in Stevanovic et al. [9], and without any signs of other infections (bacterial, viral, protozoan or fungal) in the past two years. The presence of viruses was checked according to symptoms described earlier [52] and *Varroa* infestation was kept at a low level. Frames with sealed brood were incubated at 34°C ± 1°C and newly emerged worker bees were taken, confined to six cage groups containing 40 bees in each and kept in the incubator [53]. In order to provide absolutely equal conditions for all bees from same group, and exclude the impact of all external factors (position in incubator, humidity, temperature, food amount etc.) some modifications (Fig 1) of cages presented in Williams et al. [53] were made. There were 40 individuals in each cage, needed for each treatment group (5 replicates for gene expression analyses and 5 for *Nosema* spore counting for each of the three collection times, plus 10 for mortality recording). Two independent series of experiments with essentially similar results were conducted, so the data were merged. The bees were fed ad libitum with a solution of sucrose (50% w/w). One control group was experimentally infected with *N*. *ceranae* spores (I group), the other was not (NI group), but both were fed on pure sugar syrup (without supplement). The remaining four groups were fed with sugar syrup enriched with supplement starting from day 1 (I-BW1 group), 3 (I-BW3 group), 6 (I-BW6 group) and 9 (I-BW9 group) after emerging (Table 2). All groups, except NI, were infected with *N*. *ceranae* spores. Small petri dishes (Fig 1) with the same volume of food (12 ml) were replaced daily in all cages. We have monitored the intake and noticed that the whole quantities were consumed. The supplemented sugar solutions were consumed as readily as the non-supplemented. Bees did not regurgitate the food. Dead bees were removed daily and their numbers recorded. In a preliminary investigation in both laboratory and field conditions no obvious harmful effects on bees have been observed.

**Table 2:** Design of the experiment.

Group*	Treatment with “BEEWELL AminoPlus” (day after emerging)	Sampling days (day post infection when bees were sampled for analysis)		
NI	-	3^rd^	6^th^	12^th^
I	-	3^rd^	6^th^	12^th^
I-BW1	1^st^	3^rd^	6^th^	12^th^
I-BW3	3^rd^	3^rd^	6^th^	12^th^
I-BW6	6^th^	-	6^th^	12^th^
I-BW9	9^th^	-	-	12^th^

* All groups except NI were infected with *N*. *ceranae* on day 3 after emergence

### Inoculum preparation and experimental infection

The spore solution was prepared using *N*. *ceranae*-infected bees by crushing their abdomens in distilled water. The presence of *N*. *ceranae* and absence of *N*. *apis* were confirmed with PCR following the method described by Martín-Hernández et al. [11]. Spores were counted according to Cantwell [54]. The inoculum with a minimum viability of 99% (as assessed with 4% trypan blue) was freshly prepared by mixing spores with 50% sucrose solution to obtain a final concentration of 1 x 10^6^ spores/ml. All groups, except NI, were infected with *N*. *ceranae* inoculum on day 3 after emergence according to Fries et al. [55]. To ensure that each bee ingests the full dose, individual inoculation was carried out using the protocol described in detail by Williams et al. [53].

### Bees sampling

Five bees were sampled for RNA extraction according to the following schedule: on day 3 post infection (p.i.) from groups I-BW1, I-BW3 (fed with supplement until that day) and group I; on 6^th^ and 12^th^ day after infection from all groups, with the exception of I-BW9 from which the bees were collected on day 12 (Table 2). On the same sampling days (Table 2) another five bees from each group were sampled and their abdomens were individually homogenized in 1 ml of distilled water and *N*. *ceranae* spore number per bee was estimated using a haemocytometer [54].

### Extraction of RNA and cDNA synthesis

Each single honey bee was put in a sterile 1.5-ml polypropylene microtube with 200 μl of water (PCR grade) and homogenized with a sterile disposable microtube pestle (VWR, San Francisco, CA). The total RNA was isolated from each individual honey bee with the Quick-RNA MiniPrep Kit (Zymo Research, USA), following the manufacturer’s instructions. During the extraction process the samples have passed through “in-column DNase treatment” (treatment with DNase I Reaction Mix) in order to remove any contaminating DNA. The total extracted RNA was immediately used to generate first strand cDNAs using the RevertAid^™^ First Strand cDNA Synthesis Kit (Fermentas, EU), according to the manufacturer’s instructions.

### Real-time quantitative PCR

Reverse transcription-quantitative PCR (RT-qPCR) amplification was performed using SYBR green method in a 20 μl reaction mixture with the “KAPA SYBR^®^ FAST Master Mix (2X) Universal”(KAPA Biosystems, USA), in accordance with the manufacturer’s instructions. For RT-qPCR reaction 0.2 μM of each specific primer was used. The primers used for amplification are shown in Table 3.

**Table 3:** Primers used for qRT-PCR.

Primer name	Sequence 5' to 3'	Reference
Abaecin-F	CAGCATTCGCATACGTACCA	[56]
Abaecin-R	GACCAGGAAACGTTGGAAAC	
Actin-F	TTGTATGCCAACACTGTCCTTT	[57]
Actin-R	TGGCGCGATGATCTTAATTT	
ApidNT-F	TTTTGCCTTAGCAATTCTTGTTG	[57]
ApidNT-R	GTAGGTCGAGTAGGCGGATCT	
Defensin-F	TGCGCTGCTAACTGTCTCAG	[56]
Defensin-R	AATGGCACTTAACCGAAACG	
Hymenopt-F	CTCTTCTGTGCCGTTGCATA	[56]
Hymenopt-R	GCGTCTCCTGTCATTCCATT	
VgMC-F	AGTTCCGACCGACGACGA	[57]
VgMC-R	TTCCCTCCCACGGAGTCC	

The RT-qPCR reactions were carried out in 36-well rotor using “Rotor-Gene Q 5plex”(Qiagen, Valencia, CA). The amplification was performed according to the following protocol: 95°C for 2 min followed by 40 cycles of 95°C for 20 s, 60°C for 30 s and 72°C for 80 s. Quantification of gene expression levels was done using 2^−ΔΔ*C*^_T_ method [58]. For normalization of each gene expression *β-actin* was used as an internal control gene and median value of NI group was used as a calibrator.

### Statistical analysis

Owing to homogeneity of the data (c_v_>30%), medians were determined and non-parametric tests used. The differences in the numbers of *Nosema* spores and gene transcription levels between groups and between sampling days were evaluated by Kruskal-Wallis and Mann-Whitney U test. To compare the mortality of bees between groups Kruskal-Wallis test was done. All statistical analyses were performed using software package, Statistica^®^ (StatSoft, Inc., Tulsa, OK, USA). P-values below 0.05 were considered significant.

## Results

### Presence of *N*. *ceranae* spores

**Fig 2:**
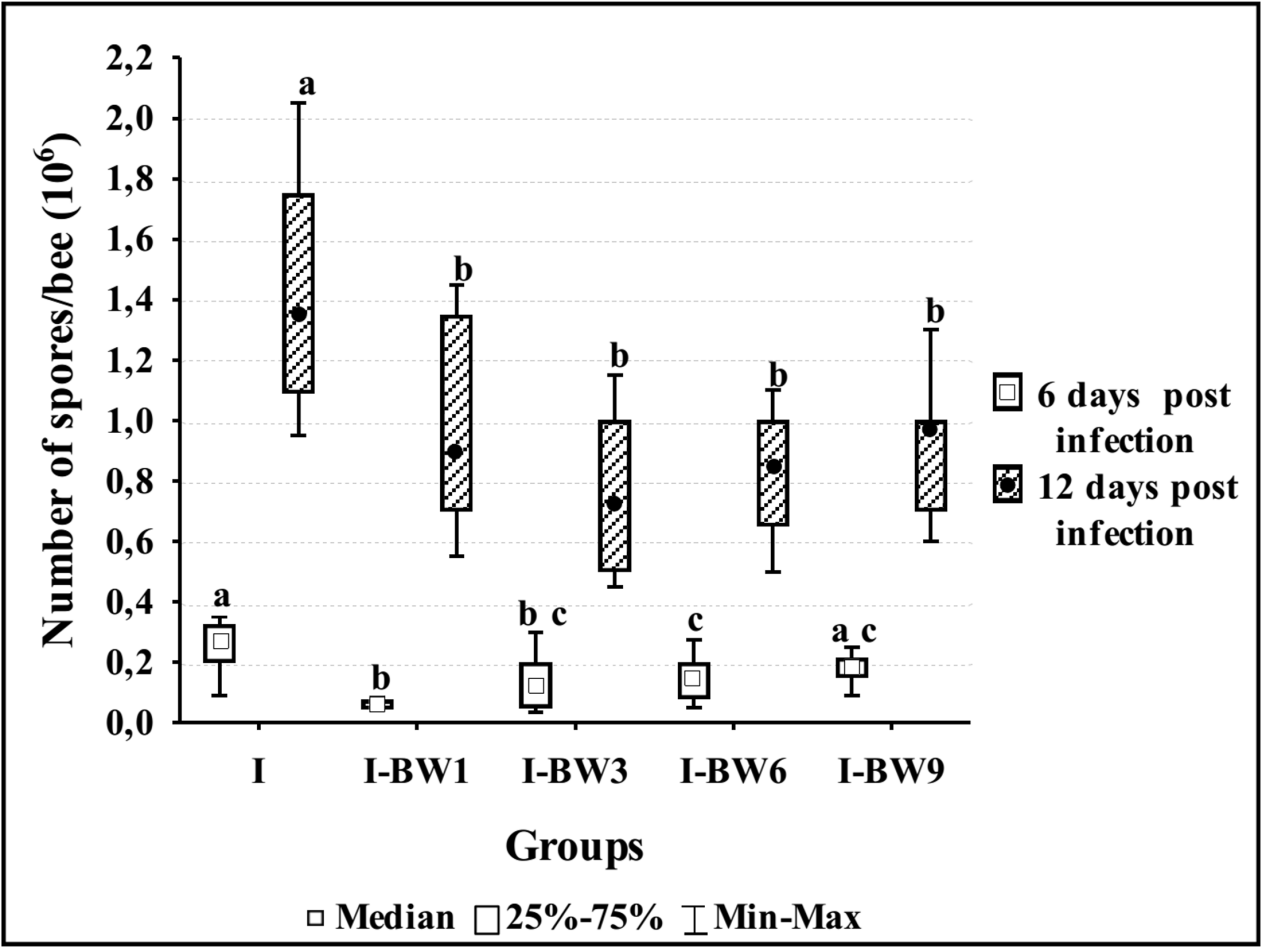
*Nosema* spore loads in control and groups treated with amino acid and vitamin complex “BEEWELL AminoPlus” on days 6 and 12 after the infection with *N*. *ceranae*. Groups were infected with *N*. *ceranae* spores on 3^rd^ day after emerging and treated with “BEEWELL AminoPlus” from 1^st^ (I-BW1), 3^rd^ (I-BW3), 6^th^ (I-BW6) and 9^th^ (I-BW9) day after emerging, while the control (I) was infected but not treated. Different letters denote significant differences between groups.

Control samples and samples collected on day 3 p.i. remained negative for *N*. *ceranae* spores over the experimental period. Kruskal-Wallis test results showed significant differences between the numbers of *N*. *ceranae* spores in groups on day 6 (H = 23.629; df = 4; p<0.001) and 12 (H = 16.809; df = 4; p = 0.002) p.i. Mann-Whitney U test results showed that the number of *N*. *ceranae* spores was significantly lower in bees collected on day 6 than in those collected on day 12 p.i. (3.781≤ z ≤3.785; p<0.001) in all groups (Fig 2).

The number of spores was significantly higher in group I than in any other on both day 6 and day 12 p.i. Mann-Whitney U test results showed that spore numbers in bees collected on day 6 p.i. were significantly higher in the control (I) than in I-BW1 (z = 3.787; p<0.001), I-BW3 (z = 2.548; p = 0.011) and I-BW6 (z = 2.316; p = 0.021) groups, while it was very close to the critical level for significance in group I-BW9 (z = 1.859; p = 0.063). On 6^th^ day p.i. number of spores was significantly lower in group I-BW1 than in I-BW6 (z = -2.919; p = 0.004) and I-BW9 (z = -3.785; p<0.001) group. Spore load on day 12 p.i. was significantly higher in I group than in I-BW1 (z = 2.235; p = 0.025), I-BW3 (z = 3.255; p = 0.001), I-BW6 (z = 3.255; p = 0.001) and I-BW9 (z = 2.919; p = 0.004) group.

### Comparison of gene expression levels between experimental groups

**Fig 3:**
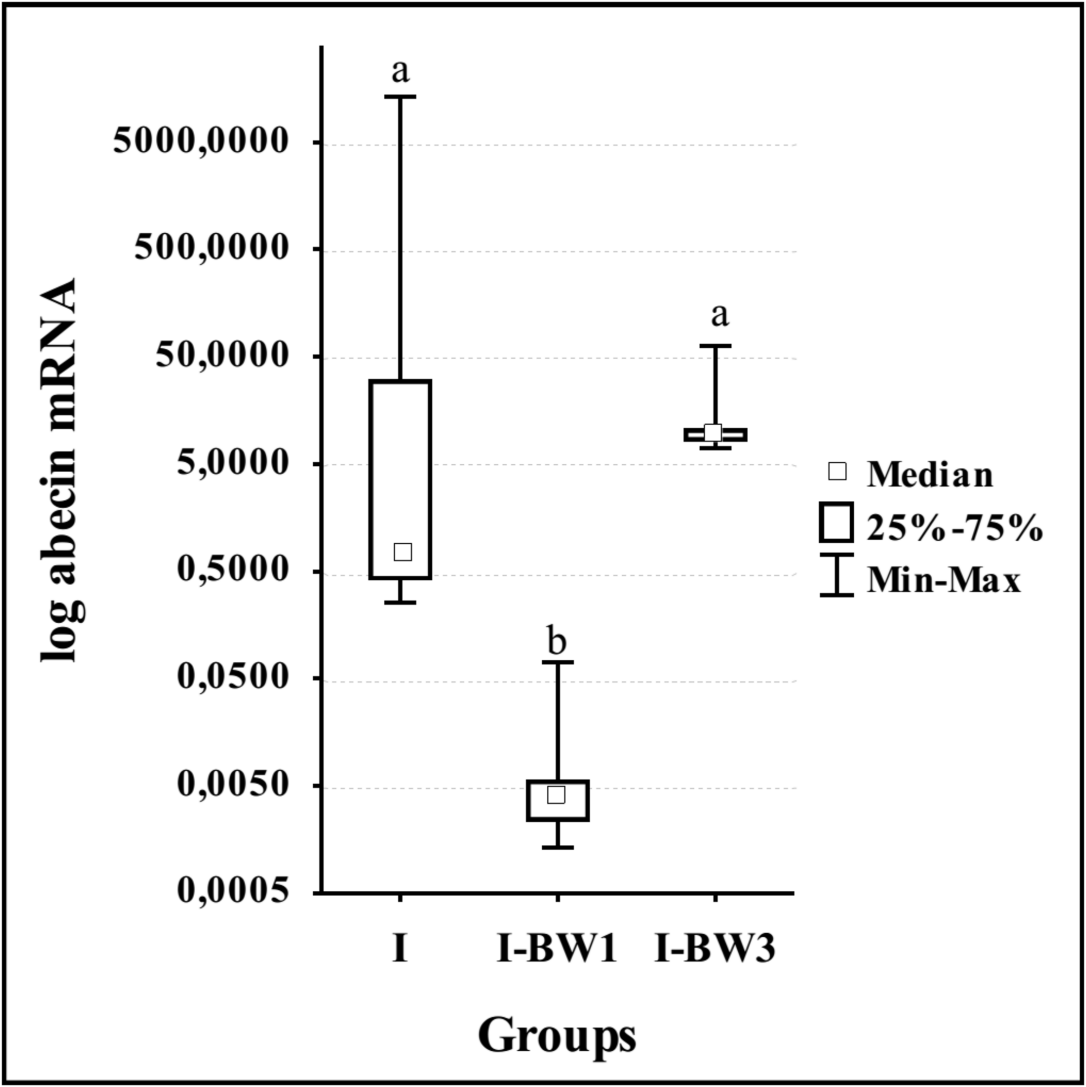
Expression levels of abaecin gene on day 3 after the infection with *N*. *ceranae* in groups treated with amino acid—Vitamin complex “BEEWELL AminoPlus”. Groups were infected with *N*. *ceranae* spores on day 3 after emerging and treated with “BEEWELL AminoPlus” from day 1 (I-BW1) and day 3 (I-BW3), while the control group (I) was infected, but not treated. Different letters denote significant differences between groups.

Mann-Whitney U test results for bees collected 3 days after infection show significant differences only in group fed with the addition of supplement from the first day (I-BW1), in which the expression of abaecin gene was significantly lower (z = 2.611; p = 0.009) than in all the others (Fig 3).

Six days after infection with *N*. *ceranae* there were no significant differences in the expression of monitored genes between bees collected from groups I, I-BW1, I-BW3 and I-BW6.

**Fig 4:**
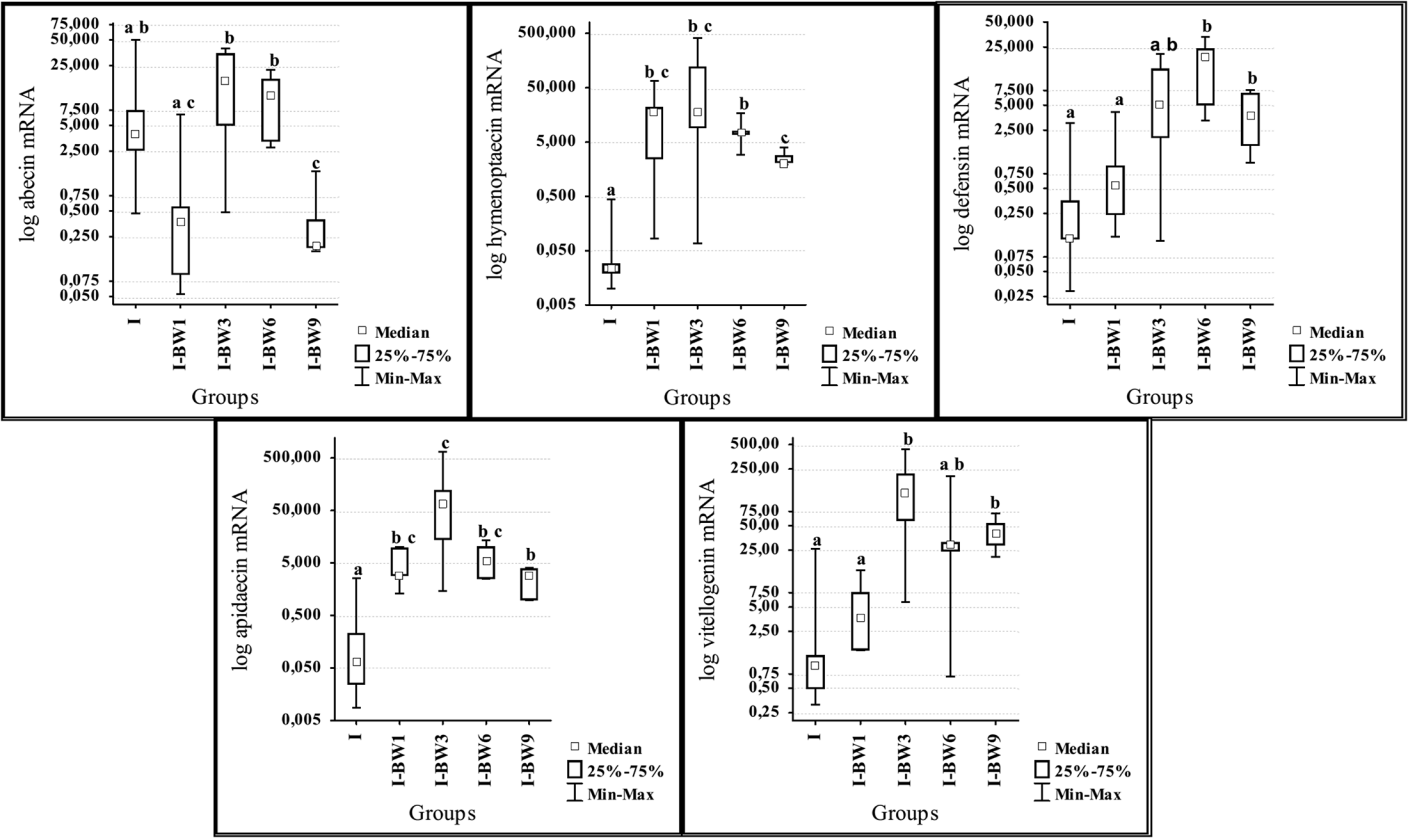
Expression levels of genes for abaecin, hymenoptaecin, defensin, apidaecin and vitellogenin on day 12 after the infection with *N*. *ceranae* in groups treated with “BEEWELL AminoPlus”. Groups were infected with *N*. *ceranae* spores on 3^rd^ day after emerging and treated with “BEEWELL AminoPlus” from 1^st^ (I-BW1), 3^rd^ (I-BW3), 6^th^ (I-BW6) and 9^th^ (I-BW9) day, while the control group (I) was infected with *N*. *ceranae* but not treated. Different letters denote significant differences between groups.

According to Kruskal Wallis test results on day 12 p.i. the levels of mRNA were significantly different for abaecin (H = 13.07; df = 4; p = 0.011), defensin (H = 12.66; df = 4; p = 0.013), apidaecin (H = 13.09; df = 4; p = 0.11), hymenoptaecin (H = 13.83; df = 4; p = 0.008) and vitellogenin (H = 13.75; df = 4 p = 0.008) (Fig 4). The expression of abaecin gene was significantly lower in bees treated from the first day (I-BW1) in comparison with I-BW3 and I-BW6, and in group treated from day 9 (I-BW9) than in bees of all other groups. Levels of hymenoptecin and apidaecin mRNA were significantly lower in the control (I) than in other groups. Defensin and vitellogenin genes were down-regulated in groups I and I-BW1 in comparison to groups I-BW6 and I-BW9.

### Gene expression through the time within the same group

**Fig 5:**
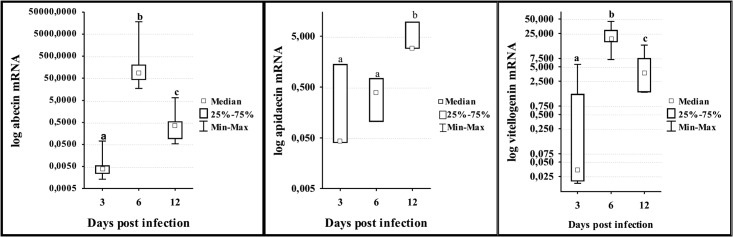
Expression levels of genes for abaecin, apidaecin and vitellogenin in control (I) and IBW-1 group on different sampling days. IBW-1 group was infected with *N*. *ceranae* spores on 3^rd^ and treated with “BEEWELL AminoPlus” from 1^st^ day after emerging, while the control (I) was infected with *N*. *ceranae* but not treated. Bees were sampled for analyses on 3^rd^, 6^th^ and 12^th^ day after the infection with *N*. *ceranae*. Different letters denote significant differences between groups.

**Fig 6:**
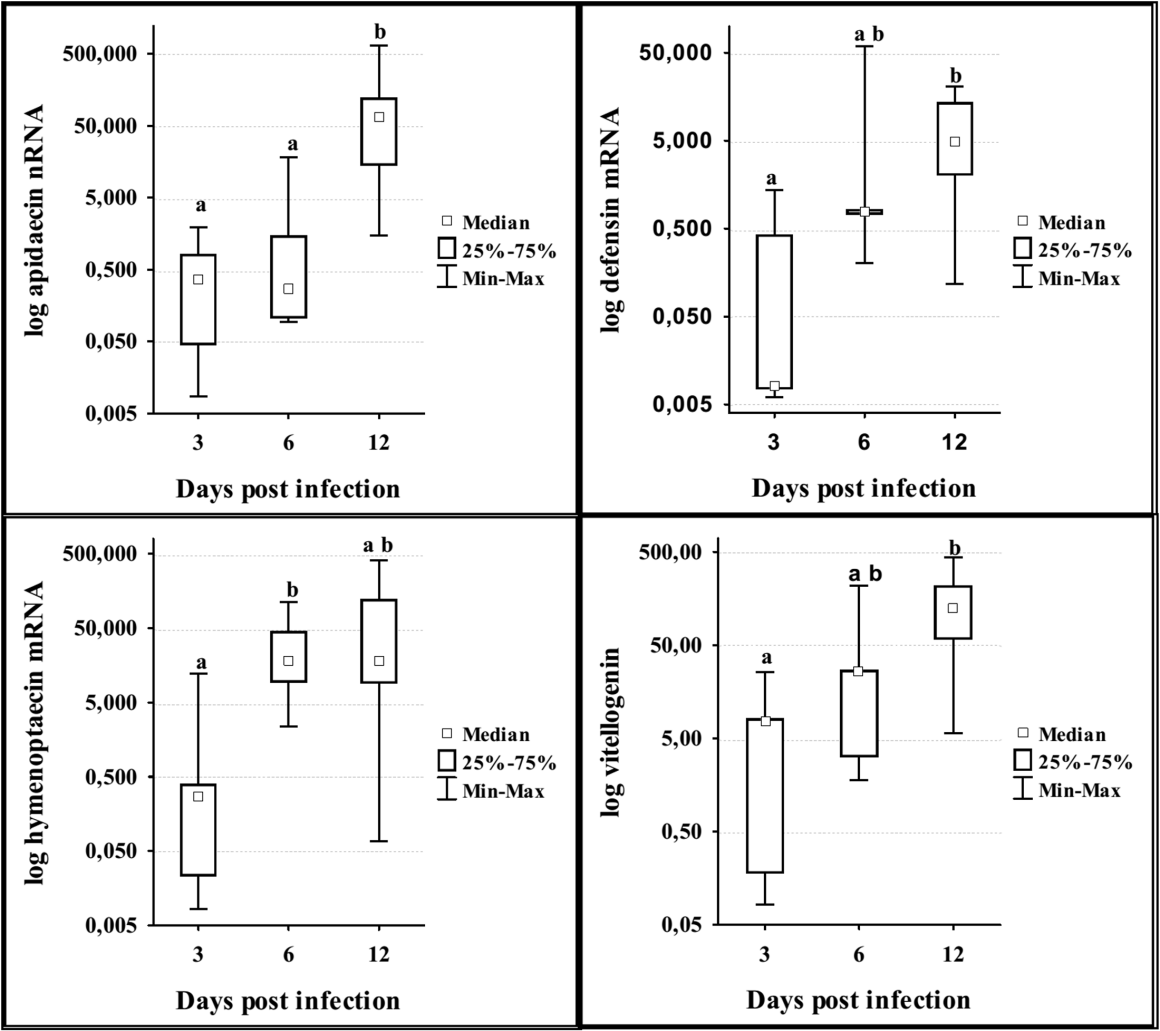
Expression levels of genes for defensin, vitellogenin, apidaecin and hymenoptaecin in group I-BW3 on different sampling days. IBW-3 group was infected with *N*. *ceranae* spores on 3^rd^ and treated with “BEEWELL AminoPlus” from 3^rd^ day after emerging. Bees were sampled for analyses on 3^rd^, 6^th^ and 12^th^ day *N*. *ceranae* post-infection. Different letters denote significant differences between collection times.

According to the results of Kruskal-Wallis test, levels of gene expression in all groups differed between sampling days (days 3, 6 and 12 p.i.), wherein the differences were very close to the critical level for significance, but only a few (Fig 5) were significant (p<0.05). Expression of abaecin in I-BW1 group varied significantly among sampling days (H = 12.02; df = 2; p = 0.002), being significantly higher in bees collected on 6^th^ day p.i. than in those collected on 3^rd^ (z = 2.611; p = 0.009) and 12^th^ day p.i. (z = 2.611; p = 0.009), and significantly lower on 3^rd^ than on 12^th^ day p.i. (z = -2.402; p = 0.016) (Mann-Whitney U test). Apidaecin gene expression levels in I-BW1 and I-BW3 group were significantly different (H = 7.58; df = 2; p = 0.023 and H = 6.86; df = 2; p = 0.032 respectively), and were higher on 12^th^ day p.i. than on other sampling days. Vitellogenin levels in I-BW1 group were significantly different (H = 10.14; df = 2; p = 0.006), being significantly lower on 3^rd^ than on 6^th^ and 12^th^ day p.i. Mann-Whitney U test revealed significant differences in the expression of defensin, vitellogenin, apidaecin and hymenoptaecin genes between bees collected on 3^rd^ and 12^th^ day in group I-BW3 in favour of 12^th^ (Fig 6).

Given that the bee mortality was very low (less than 5 bees in each cage during the whole experiment) without significant differences (H <0.001; p ≈1.000) between the groups, the safety of the supplement which was tested may be considered unquestionable.

## Discussion

BEEWELL AminoPlus" has been used by beekeepers in the Balkan countries for many years. The composition of "BEEWELL AminoPlus" is given in detail in contrast to other protein-vitamin supplements present at European and our market; it is richer in amino acids than other supplements popular in the USA [43]. However, there are no available scientific reports about "BEEWELL AminoPlus" effects on honey bees, which is why we considered important to investigate its potential to protect honey bees from induced immunosuppression. The fact that it is very rich in amino acids does not necessarily imply positive effects, as there are records of improper supplementation, although with other supplements [40,42,47,48]. It is recommended by the manufacturer that "BEEWELL AminoPlus" supplement be administered at the beginning of winter in order to promote colony health during winter. However, this may cause unwanted effects. In fact, previous experiments with natural pollen supplementation in autumn induced prolonged brood rearing, and proportionally fewer of these fall-reared workers wintered [40]. Similar side effects might happen also when artificial supplements are applied. An example of improper supplementation of bee diet with commercial probiotics and prebiotics has recently been reported: the addition of *Lactobacillus rhamnosus* (a commercial probiotic) alone or in combination with inulin (a prebiotic) induced significant increase in *N*. *ceranae* infection level and bee mortality, along with the decrease of bee immune response [59].

In our experiment spore concentration of 1 x 10^6^ spores/ml caused 100% infestation of bees, as it was reported previously [27]. The absence of infection in control bees during the whole experiment confirmed the results of Chaimanee et al. [27], who claimed that there is no *N*. *ceranae* cross-infection between cages in this type of study. Our results show that *N*. *ceranae* spores were not present 3 days p.i., but were present in all infected groups on the 6^th^ day p.i. and significantly more (p<0.001) on 12^th^ day. This could be explained by the findings of Higes at al. [60], who found that only a few ventricular epithelial cells were infected 3 days after *Nosema* infection, but the majority were infected and displayed evidence of degeneration on day 7 p.i. Spore load on 12^th^ day p.i. was significantly higher in the control (I) than in all other bees. This indicates the impact of the tested supplement on *Nosema* infection. The least difference in spore number between I and I-BW1 group on 12^th^ day p.i. suggests that it is too early to apply the supplement from the 1^st^ day of bee’s life. At the same time, down-regulation of abaecin gene was recorded on day 12 p.i. only in bees from group I-BW1. The expression of other genes was not significantly changed at that time. This finding opens a question of abaecine role during the first days of bees’ life. Such negative impacts of supplement on spore load and abecine expression recorded in I-BW1 group would not happen in hives, because young bees do not take syrup directly. Instead, they receive it by trophallaxis and use it for feeding larvae, not for their own needs [61], so they might receive only very diluted amounts of supplement. Besides, *Nosema* development is correlated with diet quality: the infection reaches higher levels in the bees fed on bee bread than in those fed on non-natural diets, i.e. carbohydrate or protein substitutes [45,62]. However, one recent study revealed that levels of *Nosema* were higher in bees fed on some commercial pollen substitutes (Ultra Bee, Bee-Pro^®^, MegaBee Winter Patty^™^, MegaBee^™^) than in those fed on pollen [63]. According to the *N*. *ceranae* spore numbers, the best period for applying supplement starts from the 3^rd^ day after emerging (bees from I-BW3 group) when bees are capable of accepting synthetic substances such as “BEEWELL AminoPlus”. Low and similar mortality in all groups (controls and fed with supplement) suggest the absence of negative impacts of “BEEWELL AminoPlus” on bees’ health and survival. No differences in mortality rate were recorded between the control and treatment groups. However, our experiment lasted 15 days, so we may assume that had it been longer bee mortality might have been higher, as it was reported by Maistrello et al. [64]. On the other hand, it is quite possible that our cage modifications (especially the insertion of a plastic mesh sink strainer into the jar lid) prevented bees to be drowned in sugar solution and enabled decrease in the mortality. The expression levels of immune-related genes on 6^th^ day p.i. were not consistent, but did not differ significantly between the groups. This finding is in accordance with the results of Higes et al. [60], who affirmed that *N*. *ceranae* achieves the peak around day 6 day p.i. However, bees collected on 12^th^ day p.i. show significant decrease in hymenoptaecin, defensin, apidaecin and vitellogenin in the control (I) in comparison with other groups. *N*. *ceranae* caused immunosuppression in I group of this study, which is in accordance with previous research [24]. However, Chaimanee et al. [27] observed down-regulation of all genes except that for vitellogenin on 7^th^ but not on 12^th^ day p.i. and explained this with differences in experimental design compared with that of Antúnez et al. [24]. Our experimental design and the one applied by Antúnez et al. [24] are comparable (although they did not analyze bees collected later than 7 days p.i.). However, our results are consistent with those of Antúnez et al. [24] with regard to imunosupression induced by *N*. *ceranae* in cage bees.

The expression of abaecin gene was variable in our study. We may assume that feeding bees with “BEEWELL AminoPlus” from the first day was too early and consequently caused significant suppression of abaecin gene. In case of 12-day old bees (supplemented from day 9), suppression of abaecin gene was probably induced by elevated *Nosema* infestation level, which is similar with hymenoptaecin, defensin and apidaecin genes. These interesting and variable levels of abaecin gene transcripts depending on supplementation time might be tested in future work. The results obtained for abaecin gene might be discussed in light of previous reports about genetic variation in expression of abaecin [65–67]. Besides, Evison et al. [68] found significant variation in expression levels of abaecin between bee patrilines. These reports enable us to justify our results obtained for abaecin gene expression, the more so we could not influence the paternity of bees in our experiment. When gene expressions were compared through the time within the same group, the levels of defensin and apidaecin were growing highly in group I-BW3, while in other groups results were not consistent. When it comes to time-dependent inconsistence in immune-related gene expression, it has been already recorded in *Nosema*-infected bees in the works of Antunez et al. [24] and Chaimanee et al. [27]. Such inconsistence in innate immune reactions may be probably explained by the permanent attempts of honey bee’s organism to maintain homeostasis. Similar to Gätschenberger et al. [69], we may suggest that modulation of gene expression in our study was the mechanism directed to achieving the balance between the urgency to activate defence reactions and the feasibility to conserve energy.

In addition, different honey bee stressors may influence DNA methylation and, consequently, the gene expression [70]. It is well known that deficiency of any of the micronutrients (folic acid, Vitamin B12, Vitamin B6, niacin, Vitamin C, Vitamin E, iron, or zinc) induce DNA damage by causing single- and double-strand breaks, oxidative lesions, or both [71]. Moreover, nutrition rich in methyl-donors may affect the DNA methylation that is implicated in social organisation of honey bees [72] and possibly in humoral, cellular and social immunity. Feeding with supplement “BEEWELL AminoPlus” containing methyl donors (e.g. methionine and vitamin B complex) may prevent DNA hypomethylation. Besides, vitamin C (as an antioxidant) in “BEEWELL AminoPlus” may contribute to the prevention of DNA oxidative lesions, possibly caused by *Nosema* parasite. Considering the difference in *Nosema* infection intensity and immune genes expression levels evidenced in our study between “BEEWELL AminoPlus”-supplemented and control bees, we hypothesize that hypomethylation of DNA and oxidative stress could contribute to immunosuppression. Further investigations, both laboratory and in-hive (for oxidative stress research) and in the field (for gene expression assessment) are necessary to confirm this hypothesis.

Some previous results revealed the beneficial impact of nutrition on bee health, especially a link between protein nutrition and immunity, longevity, survival of bees and their defence against pathogens [32–34,45,51]. Our results indicate a similar impact on bee health exerted by “BEEWELL AminoPlus”. Its usage as an immunoprotective supplement for *Nosema*-infected colonies is absolutely advisable because treatments with fumagillin, although effective, could be problematic to bee health, quality of hive products and health of consumers [73–75].

These results of modified expression of some immune-related genes caused by “BEEWELL AminoPlus” enable us to hypothesize that this supplement (very rich in amino acids and vitamins) could influence the honey bee immunity. The hypothesis is in concordance with the previous discoveries of a direct link between protein nutrition and honey bee immunity, precisely betweenthe pollen quality and diversity, and bee health, individual and social immunity, by means of influencing their physiology, longevity and tolerance to pathogens and parasites [32,34,39,44,51]. In fact, pollen upregulates genes that code for antimicrobial peptides (lyzozyme-2, lyzozyme-3 and defensin-1), which contribute to individual immunity [34] as well as those affecting longevity (like genes that code antioxidants vitellogenin and superoxide dismutase) and also activates nutrient-sensing and metabolic pathways in individual bees [34]. Compared with monofloral, polyfloral diets induced higher activity of glucose oxidase (GOX), the parameter of social immunity [32]. Pollen richest in proteins and lipids (polyfloral and protein-richest monofloral pollen) significantly increased the expression of genes for vitellogenin and transferrin and the development of hypopharyngeal gland acini [44] confirming the importance of pollen quality for nurse bee physiology, immunity, and the survival of *Nosema*-parasitized bees [44]. The importance of pollen diversity for the bee’s immunocompetence has been supported in the long-term study of Antúnez et al. [51]. All these data and the fact that bees would never use integral plant proteins, but will degrade them to amino acids and other biologically-active substances lead us to presume the possible mechanism of „BEEWELL AminoPlus”to modify and/or increase the expression of some immune-related genes. The tested supplement is very rich in amino acids that are easily absorb, which makes it possible to achieve similar effects as fermented polyfloral diet or bee bread and even better effects than many other artificial protein diets [32,44,45,51]. To confirm this hypothesis, further in-hive research and comparison with bee bread is required.

This study demonstrated the negative impact of *N*. *ceranae* on bee health as reported previously [4,10,13–16,18,76,77]. Our results indicate that tested supplement “BEEWELL AminoPlus” has potential to modify the expression of immune-related genes in honey bees compromised by *N*. *ceranae* infection. The supplement showed best efficacy when applied simultaneously with *Nosema* infection (I-BW3 group) suggesting early spring as most convenient period for its application because *Nosema* spore load in the hive is highest in this period [76,78–80]. Nevertheless, additional testing of the supplement effects in hive experiment is necessary to confirm this hypothesis.

## Supporting information

S1 Text Summary of product characteristics. (PDF)

S1 File A schematic plan of the experiment. *Treatment groups (I-BW1, I-BW3, I-BW6 and I-BW9) were supplemented with “BEEWELL AminoPlus” starting from 1^st^, 3^rd^, 6^th^ and 9^th^ day after emergence, respectively. All treatment groups and the control group (I) were infected with *N*. *ceranae* spores on day 3 after emergence. The non-infected control (NI) was neither infected, nor supplemented. **Out of 10 bees sampled, 5 were used for gene expression analysis and 5 for *Nosema* spore count calculation.
(PDF)
