# Supplementary material for: Dietary amino acid and vitamin complex protects honey bee from immunosuppression caused by Nosema ceranae
Source: PLoS One. 2017 Nov 8;12(11):e0187726. doi: 10.1371/journal.pone.0187726 (PMC5678887; doi:10.1371/journal.pone.0187726)
Supplement: S1 Text — (PDF) [file pone.0187726.s001.pdf]

SUMMARY OF PRODUCT CHARACTERISTICS

1. NAME OF THE VETERINARY MEDICINAL PRODUCT
BEEWELL AminoPlus

2. QUALITATIVE AND QUANTITATIVE COMPOSITION
Composition per litre
Tryptophan 2.000 mg
Hydroxypyroline 15.000 mg
Glutamic acid 15.000 mg
Asparagine 7.000 mg
Phenylalanine 1.500 mg
Hydroxylysine 1.200 mg
Tyrosine 600 mg
Proline 15.000 mg
Alanine 15.000 mg
Histidine 1.000 mg
Glycine 30.000 mg
Serine 5.000 mg
Leucine 6.000 mg
Valine 5.500 mg
İsoleucine 5.400 mg
Threonine 3.000 mg
DL-Methionine 2.500 mg
L-Lysine 10.000 mg
Vitamin K3 1.500 mg
Vitamin-C 3.000 mg
Calcium D-pantothenate 1.200 mg
Niacin 2.400 mg
Vitamin B12 1 mg
Vitamin B6 200 mg
Vitamin B2 800 mg
Vitamin B1 200 mg
Vitamin E 280 mg
Vitamin D3 270.000 i.u
Vitamin A 1.800.000 i.u
For a full list of excipients, see section 6.1.

3. PHARMACEUTICAL FORM
Oral Suspension

4. CLINICAL PARTICULARS

4.1 Target species
Honey bees

4.2 Indications for use, specifying the target species
Supplement for honey bees
To treat and/or prevent: Nosema, Foulbrood (American or European) Stone, Varroa (in winter time)
To improve performance and fertility

4.3 Contraindications
None known

4.4 Special warnings for each target species
None.

4.5 Special precautions for use
Special precautions for use in animals
Apply the product in early morning or evening.
Avoid or direct sunlight during application.
Do not use more than 2 ml / 1 liter of syrup as overdosage my cause extra deaths .
Do not use more than recommended dosages and intervals.

Special precautions to be taken by the person administering the veterinary medicinal product to animals
None known.

4.6 Adverse reactions (frequency and seriousness)
None known.

4.7 Use during pregnancy, lactation or lay
Not applicable

4.8 Interaction with other medicinal products and other forms of interaction
None known.

4.9 Amounts to be administered and administration route
Oral with syrup.
Nosema: Add 1 ml per 1 liter of syrup for 2 days .
Repeat the application when the symptoms are seen again.
Foulbrood:
Add 1 ml per 1 liter of syrup for 3 days .
Repeat the application after one week .
Stone :
Add 1 ml per 1 liter of syrup for 3 days .
Repeat the application after one week .
Varroa in winter time :
Add 1 ml per 1 liter of syrup for 2 days .
Repeat the application after one week .
The product is not effective against varroa in summer time .

Performance and fertility :
In order to prepare the colonies for spring add 1 ml per 1 liter of syrup for 2 days . Repeat the application after 10 days.
Second harvest during season
In order to promote the colonies for second harvest of a different area or source add 1 ml per 1 liter of syrup 10-15 days before harvest.
Safe and strong winter time
In order to promote the colonies to be healthy during winter add 1 ml per 1 liter of syrup At the beginning of winter season.

4.10 Overdose (symptoms, emergency procedures, antidotes), if necessary
Do not use more than 2 ml / 1 liter of syrup as overdosage my cause extra deaths .
Do not use more than recommended dosages and intervals.

4.11 Withdrawal period(s)
NIL

5. PHARMACOLOGICAL PROPERTIES
The product consists of a combination amino acids and vitamins for use as supportive maintenance therapy and prevention in conditions of stress and diseases and to improve fertility and performance.

6. PHARMACEUTICAL PARTICULARS

6.1 List of excipients
Purified water
Sorbitol q.s

6.2 Incompatibilities
None known

6.3 Shelf life
Shelf-life of the veterinary medicinal product as packaged for sale: 3 years

6.4. Special precautions for storage
Do not store above 25 C.
Do not freeze.
Protect from light.
Discard unused material.

6.5 Nature and composition of immediate packaging
30 ml HDPE syringe.

6.6 Special precautions for the disposal of unused veterinary medicinal product or waste materials derived from the use of such products
Any unused veterinary medicinal product or waste materials derived from such

veterinary medicinal products should be disposed of in accordance with local requirements.

7. MANUFACTURER
Provet Genom Biotechnology Laboratory
Ivedik OSB 30. Cd. 530 Sk no.6-8 Yenimahalle Ankara Turkey

Local authorized representative and distributor: ANISANA doo, Blagoja Parovica 91, Belgrade, Serbia.
