# Supplementary figures and images for: Dietary amino acid and vitamin complex protects honey bee from immunosuppression caused by Nosema ceranae
Source: PLoS One. 2017 Nov 8;12(11):e0187726. doi: 10.1371/journal.pone.0187726 (PMC5678887; doi:10.1371/journal.pone.0187726)

## A schematic plan of the experiment

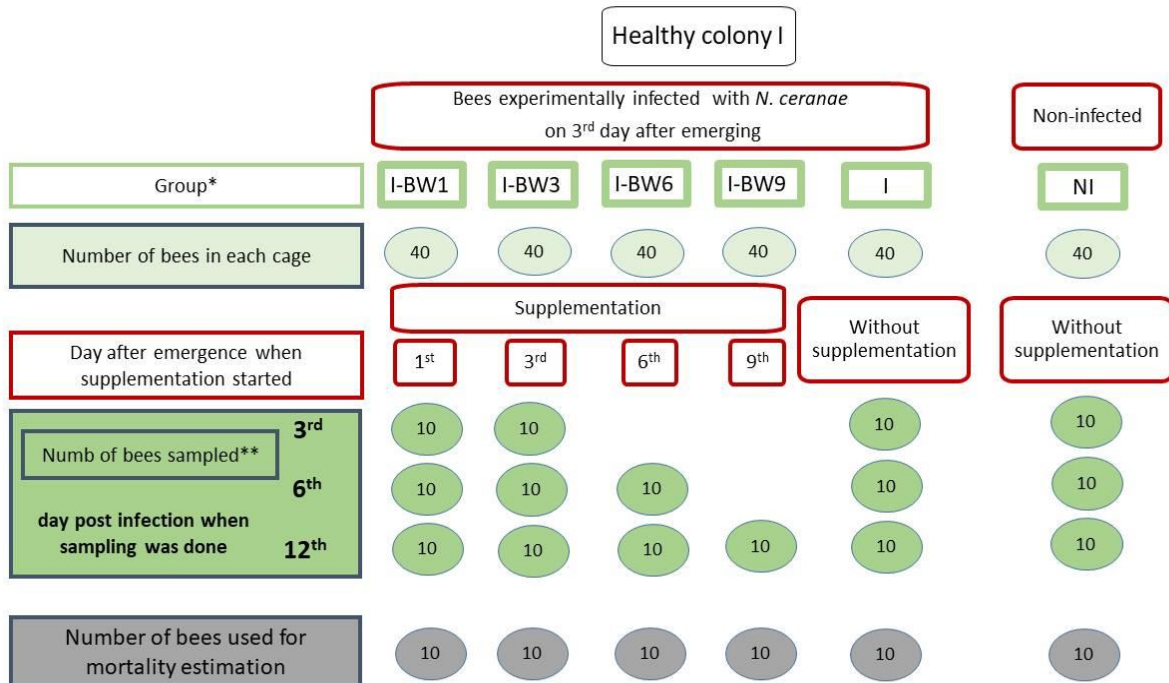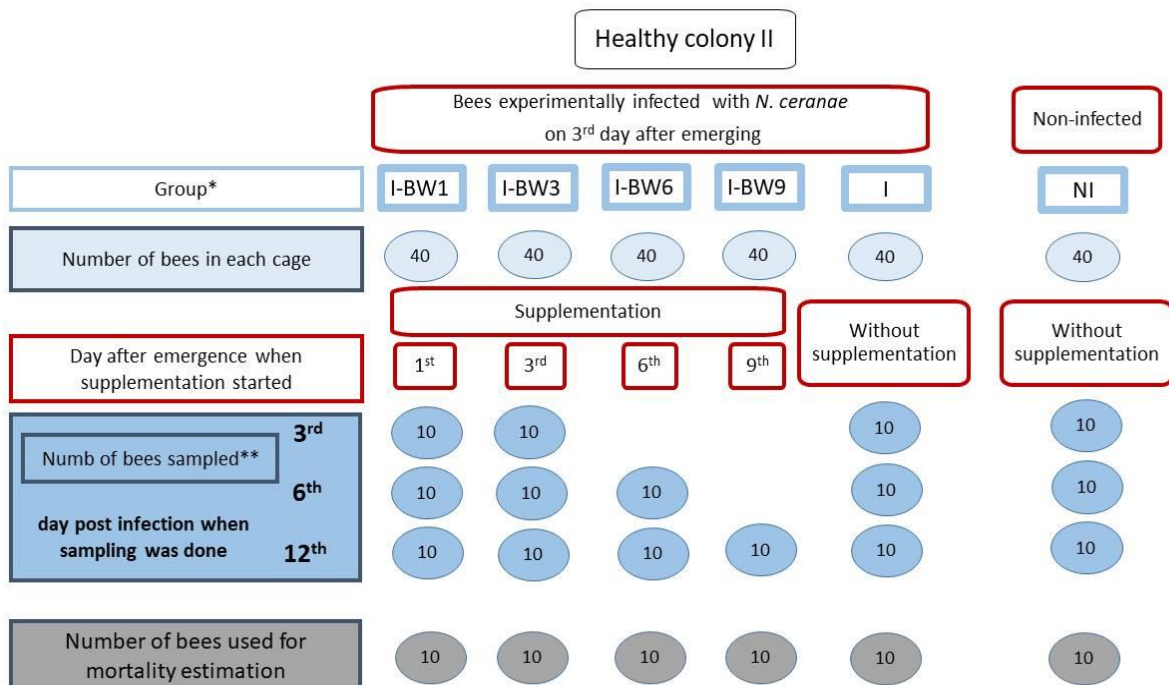

Supplement: S1 File — *Treatment groups (I-BW1, I-BW3, I-BW6 and I-BW9) were supplemented with “BEEWELL AminoPlus” starting from 1st, 3rd, 6th and 9th day after emergence, respectively. All treatment groups and the control group (I) were infected with N. ceranae spores on day 3 after emergence. The non-infected control (NI) was neither infected, nor supplemented. **Out of 10 bees sampled, 5 were used for gene expression analysis and 5 for Nosema spore count calculation. (PDF) [file pone.0187726.s002.pdf]
